# Performance of Medium-Rise Buildings with Reinforced Concrete Shear Walls Designed for High Seismic Hazard

**DOI:** 10.3390/ma16051859

**Published:** 2023-02-24

**Authors:** Claudio Alarcón, Álvaro López, Juan Carlos Vielma

**Affiliations:** School of Civil Engineering, Pontificia Universidad Católica de Valparaíso, Valparaíso 2340000, Chile

**Keywords:** non-linear analysis, RC shear wall structure, collapse limit state, incremental dynamic analysis, fragility curves

## Abstract

This work has evaluated the collapse fragility of a typical Chilean building for residential use, structured based on shear-resistant RC walls and inverted beams arranged along its entire perimeter, using the incremental dynamic analysis (IDA) for the evaluation of its structural behavior, using for this the 2018 version of the SeismoStruct software. This method evaluates the global collapse capacity of the building from the graphical representation of its maximum inelastic response, obtained through a non-linear time–history analysis, against the scaled intensity of a set of seismic records obtained in the subduction zone, thus creating the IDA curves of the building. The processing of the seismic records is included within the applied methodology to make them compatible with the elastic spectrum of the Chilean design, achieving an adequate seismic input in the two main structural directions. In addition, an alternative IDA method based on the elongated period is applied to calculate the seismic intensity. The results of the IDA curve obtained with this procedure and the standard IDA analysis are analyzed and compared. The results show that the method relates very well to the structure’s demand and capacity and confirms the non-monotonous behavior exposed by other authors. Regarding the alternative IDA procedure, the results indicate that the method is inadequate, failing to improve the results obtained by the standard method.

## 1. Introduction

Chile is in one of the world’s highest active seismic zones. In addition, Chile has a demanding seismic-resistant design standard that states that structures must be designed so that they do not suffer damage of any kind during seismic events that occur frequently, that is, several times during the period of useful life (50 to 70 years) of the building. Nevertheless, it also establishes that the structures can suffer damage and even may be demolished after the earthquake. Currently, the Chilean seismic design code [1] uses the traditional force-based method for building design. This method is widely used but has several limitations that do not allow the designer to adequately control the performance, especially when strong earthquakes occur [2].

On the other hand, to better understand the structural implications of rarer or more severe seismic movements, studying the response within the non-linear range provides relevant information about the structure’s performance. This includes, for instance, the location and deformation demand in critical sections of the structural system, the resistance that the elements must have that are expected to remain elastic, the accelerations in each story that are related to the damage in the content, and the displacements that the building must be capable of holding [3].

Tremendous computational advances have facilitated the appearance of increasingly precise and refined analysis methods, but at the same time they have become more complex. Thus, state of the art analysis has progressively gone from elastic static analysis to dynamic, non-linear static, and finally to non-linear dynamic. In the latter case, the usual practice has been to conduct these analyses using one or several accelerograms. In the latter case, the standard practice has been to analyze with one or several records, producing one or several “single point” analyses, used mainly to check the performance of the designed structure [4,5,6,7]. On the other hand, methods such as the static non-linear Pushover (SPO) analysis or the capacity spectrum method [8] offer, by appropriate scaling of the static force pattern, a “continuous” picture as the full range of structural behavior is investigated, from elasticity to yielding and finally collapse, thus greatly facilitating the understanding of the behavior of structures subjected to earthquake action [9,10].

By analogy, going from a single static analysis to a static incremental one (pushover), a single time–history analysis is extended to an incremental one, where the seismic demand is scaled up. The concept has already been mentioned by Bertero [11] a few decades ago and reflected in various ways in other studies [12,13,14,15,16,17].

Thanks to constant computer improvements, procedures have been developed that even make it possible to evaluate potential victims from a partial or total collapse of a building. To this end, this study presents the development of the collapse fragility curves, obtained from the non-linear dynamic analysis of a typical Chilean building, to relate the probability of collapse as a function of a measure of intensity associated with the seismic demand. The incremental dynamic analysis (IDA) proposed by Vamvatsikos and Cornell [18,19] and adopted by FEMA P-58-1 [20] is used to determine the global collapse capacity of the structure. This methodology produces a distribution of results at different intensities used to generate collapse fragility curves [21,22,23].

One of the main characteristics of buildings in Chile consists of their structure based on reinforced concrete (RC) shear walls. The effect of shear on the seismic performance of buildings has received much attention during the past decades through the work of [24] and [25], with particular attention on improving the seismic resistance of RC structures. The work of [24] describes the addition of fiber-reinforced polymer to the diaphragms to increase their shear capacity. At the same time, [25] focuses on the innovation of shear walls by incorporating a self-centering balancing mechanism to improve their seismic performance in terms of lateral resistance, deformation capacity, and damage minimization.

On the other hand, the evaluation of buildings’ seismic performance is based on information from experimental tests and numerical analysis [26,27,28,29,30]. Within the latter, the works carried out to evaluate the behavior of shear wall structures are relevant, since they allow us to know in detail the causes that have permitted this typology to have had an adequate performance during strong earthquakes that occurred in recent times. It should be noted that many researchers have chosen the fragility curves obtained through non-linear analysis to have a quantification of the probability of exceedance of certain damage states, especially the collapse damage state [31,32,33,34,35,36,37]. However, there needs to be more relevant information on the fragility curves of this type of building in Chile, allowing us to understand their adequate performance.

Currently, in Chile, no regulations indicate and control the guidelines for seismic vulnerability studies. There are only regulations for the seismic design of buildings [1], but where the concept of seismic vulnerability (i.e., fragility curves) is not included [38,39]. For the above reason, this study aims to evaluate the non-linear dynamic behavior of a half-height Chilean RC building by determining its collapse fragility curves. It also gives rise to a need to contribute in some way to the knowledge of new seismic vulnerability analysis methodologies, especially given the degree of progress of more rigorous numerical models that allow the appropriate analysis of complex structural typologies such as those buildings whose primary resistant system is based on RC shear walls [40,41,42,43,44,45]. This study begins by explaining the fundamental concepts necessary to understand the IDA methodology, followed by the theory of the procedure, the definition of the analysis parameters, and the step by step procedure to obtain the collapse fragility curves according to FEMA [20]. Then, the building under study is described, the acceleration records selected as seismic demand are processed, and the calculations of maximum structural responses are presented. Subsequently, the criteria considered in each non-linear response history analysis are explained, and the results are illustrated in IDA curves. Afterward, an alternative IDA procedure is carried out to evaluate, analyze and compare the results obtained from this method with the standard IDA procedure. Finally, the collapse fragility curves of the structure are obtained, and the results are analyzed, allowing appropriate conclusions and future recommendations to be drawn.

## 2. Methodology for the Seismic Assessment

Dynamic incremental analysis corresponds to a non-linear response history analysis and is the most reliable method for obtaining collapse fragility curves [19]. This procedure can be carried out for any building to estimate the different median capacities and requires repeated analysis for multiple acceleration records. The results allow the development of a function with a higher level of accuracy since it considers the true record-to-record variability in each response.

Currently, the international community recognizes this type of analysis as the most sophisticated and accurate for assessing the vulnerability of structures subject to strong ground movements. However, it has the disadvantage that it is a type of analysis with a high computational cost, which makes its use very limited to the cases that justify it [10].

According to Vamvatsikos and Cornell [19], dynamic incremental analysis is an integral method to extract the conditional distribution of structural response (e.g., maximum drifts or maximum accelerations of each building story) from different levels of seismic demand. This procedure is achieved by subjecting a structural model to a non-linear time–history analysis under a set of accelerograms from other seismic events, which are scaled increasingly for each measurement of intensity, thus increasing the energy of the record to force the structure from elasticity to global dynamic instability or collapse.

Regarding the procedure, its amplitude must be increased for each selected accelerogram. The non-linear response history analysis is carried out until some collapse condition is achieved (e.g., story drift exceeding a collapse limit) or a numerical instability occurs (e.g., a significant increase in story drift associated with a slight rise in spectral acceleration) [46,47,48]. For each analysis, the maximum response of the structure is recorded, which must be a parameter consistent with the selected damage indicator. Then, it is plotted on a graph that relates this result to the indicator parameter of the damage intensity measure (e.g., maximum inter-story drift vs. spectral acceleration of the first mode $S_{a}\left(T_{1}\right)$). Once the discrete points of the dynamic incremental analysis have been obtained for a given record, the entire IDA curve can be approximated without needing additional analysis and using a piecewise linear approximation or upper spline interpolation. A realistic interpolation that accurately represents the actual IDA curve can be generated [19]. This process is repeated for each record selected as seismic demand to obtain IDA curves.

The probability of suffering a structural collapse is expressed as a smooth lognormal distribution that fits the fraction of ground motions at each level of intensity that causes a failure, defined by a mean value ${\hat{S}}_{a}\left(\bar{T}\right)$ and dispersion β. This value is the mean collapse capacity and corresponds to the value of the spectral pseudo-acceleration at which 50% of the ground motion pairs produce collapse predictions. The basic steps of the procedure to obtain the fragility curves are as follows:

1. Build a mathematical model that represents the structure.
2. Select an appropriate set of ground motion pairs representing seismic events that may result in the structure’s collapse, considering the site’s seismic risk.
3. Scale the pairs of ground motions such that the pseudo-spectral acceleration in the fundamental period of the structure, $S_{a}\left(T_{1}\right)$, for each pair has a value low enough that the inelastic response is negligible.
4. Analyze the mathematical model for each scaled ground motion pair and determine the maximum value of the drift that occurs among all its levels.
5. Increase the intensity of the effective spectral pseudo-acceleration in the fundamental period, $S_{a}\left(T_{1}\right)$, to which each ground motion pair is scaled.
6. Analyze the structure for each incrementally scaled ground motion pair and record the maximum value of the ground drift.
7. Repeat steps 5 and 6 above for each pair of ground motions until the analysis predicts a collapse of the structure.
8. Determine the value of the spectral pseudo-acceleration $S_{a}\left(T_{1}\right)$ in which 50% of the ground motion pairs produce collapse predictions. This value of $S_{a}\left(T_{1}\right)$ is taken as the mean collapse capacity, ${\hat{S}}_{a}\left(\bar{T}\right)$.
9. Determine a scattering for the collapse fragility.
10. Three mechanisms of collapse are distinguished in this methodology. One is a lateral failure or lateral dynamic instability, characterized by the loss of lateral stiffness and development of P-delta instability. Another collapse mechanism is loss of vertical bearing capacity in resistant gravity or seismic components due to earthquake-induced drift. Finally, collapse can be defined as exceeding non-simulated failure criteria, such as maximum force or deformation where components can no longer resist the load reliably.

The global collapse of a structure is related to the value of IM or DM where dynamic instability is observed. As defined by FEMA in [49,50], the global collapse capacity is a union of the rule based on 20% IM slope and a rule based on the MD of $\delta_{max}$ = 10% where $S_{a}\left(T_{1}\right)$ and $\delta_{max}$ are the IM and DM of choice. If either of the two rules is obtained, the capacity is defined, meaning that the 20% local slope detects impending collapse. In comparison, the 10% cap protects against extreme values of $\delta_{max}$, indicative of regions where the model may not be reliable [18,19].

This work seeks to apply seismic motions that occurred in the subduction zone, which better represent the seismic demand that will affect the structure under study. To this end, a series of seismic records must be selected, considering the main events of different seismogenic sources that make up the Chilean seismic hazard. According to FEMA P-58-1 [20], around 20 pairs of suitable seismic records should be used to obtain reliable estimates of collapse fragility. Nonetheless, for some structures, it may be less. For instance, 11 pairs of registers, each rotated 90 degrees to produce a total of 22 sets of movements, were found to be sufficient.

**Figure 1:**
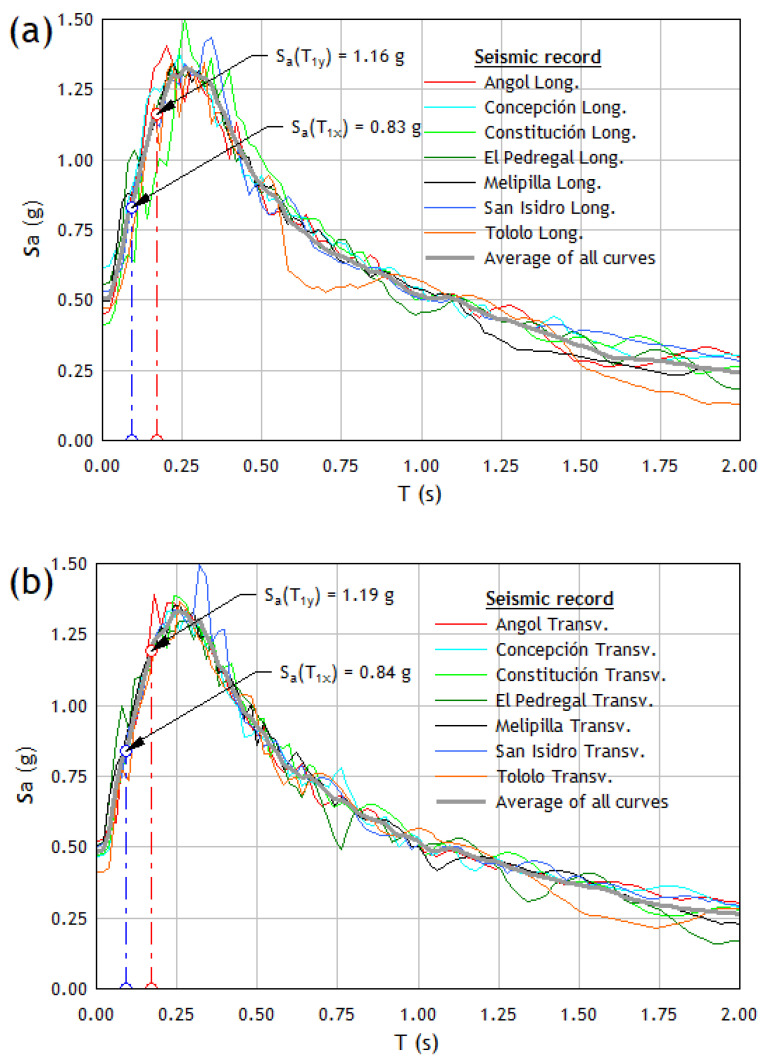
Elastic response spectrum of the chosen acceleration records selected: (**a**) Longitudinal (**b**) Transverse.

Based on the criteria detailed above, seven accelerograms or pairs of seismic records compatible with the Chilean response spectrum were selected, corresponding to three of the strongest earthquakes in Chile in the last 50 years. The accelerograms were obtained from the following databases: Strong-motion Virtual Data Center (VDC) and Center for Engineering Strong Motion Data (CESMD). Table 1 shows the details of three earthquakes considered for the dynamic incremental analysis, including the date of occurrence, moment magnitude ($M_{w}$), coordinates of the epicenter, and the station where the recording took place. Figure 1 shows the elastic spectra of the acceleration response for the two orthogonal components of each of the seven seismic records paired with the elastic spectrum of the design of the structure, a procedure for which the SeismoSignal program [51] has been used.

## 3. Case Studied

The building is located in Quillota, in the Valparaíso region of Chile. It corresponds to a typical Chilean building for residential use, designed according to the current Chilean regulation NCh433 and verified by linear analysis. This building has been selected as representative of the seismic-resistant design applied to RC buildings in Chile due to the distribution of resistant members typical of a residential building, in which the central corridors define the orientation of the walls in the longitudinal direction. Another important aspect is the intermediate height of the building, characterized by thin shear walls with reinforcing steel as per design, making it like buildings that are part of a significant percentage of the stock of buildings in Chile.

**Figure 2:**
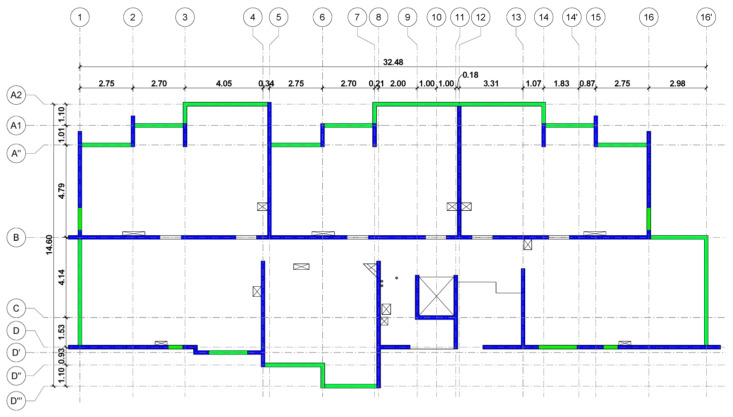
Typical plan view of the building, with the shear walls marked in blue and the beams in green.

The supporting structure of the building, which is considered representative of Chilean seismic engineering, consists of shear-resistant RC walls. Practically 77% of the buildings built in the country from 1940 onwards support gravity and lateral loads with RC shear walls, and most have residential use with a height between 5 and 25 stories. This type of structure is commonly called “fishbone” (see Figure 2), which is characterized by presenting a typical plan with a longitudinal central corridor with shear walls and transversal walls that separate the apartments of the building and the interior rooms. The latter extends orthogonally to the corridor walls, reaching outside the building, creating a fishbone shape [52].

**Figure 3:**
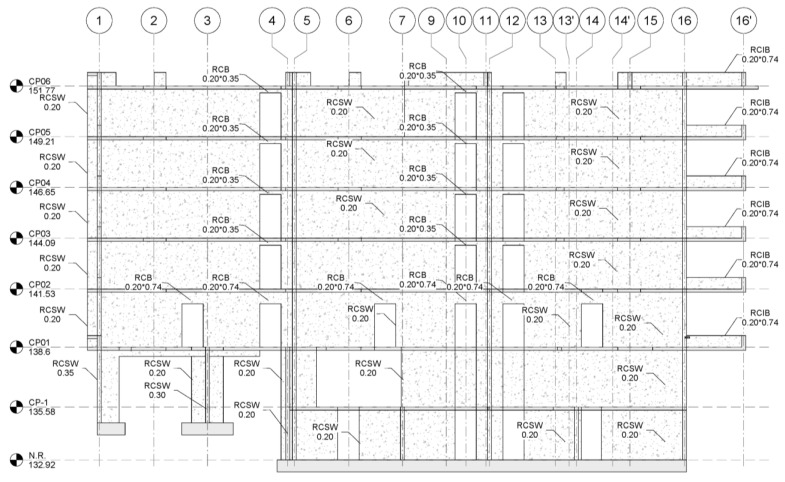
Elevation view of the section of the building in axis B.

**Figure 4:**
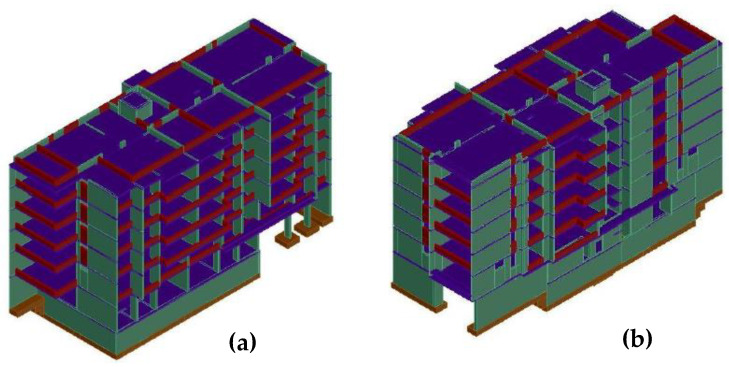
Axonometric perspective (**a**) Northeast and (**b**) southeast.

The walls are evenly distributed in the two main directions, which makes the building very stiff and can withstand horizontal seismic actions well. On the other hand, the relationship between the area of walls that act actively in the direction of their main length and the area on the floor plan, that is, the density of walls, is relatively large in constructions in Chile compared to buildings of similar height in seismic regions of the US. Shear wall density most commonly ranges from 1.5% to 3.5% in each direction, with a mean value of 2.8%, a characteristic that has remained almost constant over time [53,54,55]. In addition to shear-resistant walls, another typical feature of Chilean structures is the use of inverted beams, which are intended to stiffen the building by keeping the walls connected. The foundations consist of continuous footings that allow the direct support of the shear walls. The reinforcement of the structural elements has been carried out following the usual Chilean practice; for more details, the reader can refer to the recently published work [39,56]. Regarding the inverted beams, they are arranged around its entire perimeter, consisting of six floors and a basement (see Figure 3 and Figure 4). The first two floors are used as commercial businesses, and the rest are apartments for civilian housing.

Materials properties, structural elements sizes and reinforcement detailing, and description of the various phases involved in developing the structural model of the reference structure for the case study can be found in [39,56]. Three-dimensional non-linear finite element modeling was carried out using SeismoStruct [57]. Walls and beams were modeled using force-based, Euler–Bernoulli fiber beam–column elements with five integration points that account for the spread of plasticity along the length of the element [58,59,60]. A section discretized into unconfined concrete, confined, and steel fibers was located at each element’s integration point. Uniaxial material models with a non-linear constitutive relationship were assigned to the fibers. For concrete, Chang–Mander [61] non-linear concrete material model was used, which emphasizes the transition of the stress–strain relation upon crack opening and closure compression and tension with a cyclic behavior like that in compression. The Menegotto–Pinto steel material model, based on Menegotto and Pinto’s constitutive model [62], was used for reinforcing steel. Beams were provided with rigid-end zones to account for the stiffness of the wall–beam joint. The seismic weights were distributed at the wall–beam or wall–slab joints and automatically transformed into seismic masses to perform the dynamic analyses.

## 4. Results and Discussion

Table 2 shows the periods, frequencies, and percentage of participating effective mass for the first ten modes of the structure, according to the three directions (X, Y, Z). The first mode is predominantly longitudinal in the Y direction (the less stiff direction of the building). Still, there is a non-negligible percentage of participating effective mass for the rotation around X and Z. Therefore, the fundamental period of the building in the Y direction corresponds to the first mode of vibration of the structure. On the other hand, the third mode is translational along the X-axis, with a relevant rotation component around the Y-axis. Therefore, the fundamental period of the building in the X direction corresponds to the third mode of vibration of the structure.

The fundamental periods of vibration in each principal direction of the structure shown in Table 2 produce high spectral ordinates for both the longitudinal and transverse components of the seismic records (see average curves in Figure 1). This feature guarantees that the seismic records applied will introduce high acceleration values to the structure that will allow, on the other hand, to apply the dynamic incremental analysis appropriately.

**Figure 5:**
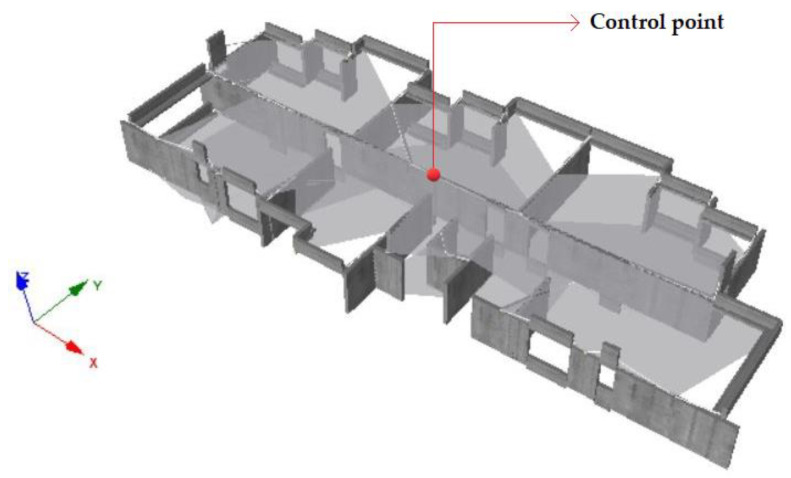
Example of displacement control point.

This work proposes the maximum inter-story drift $\delta_{max}$ as a parameter to measure the structural response under seismic action. This variable is computed by determining the total horizontal displacement of each building level and divided by the story height. A control point or master node representing each floor’s response was identified in the center of the slab. Figure 5 details the displacement control point corresponding to the third story.

Once the control points in each of its floors have been identified in the numerical model, it is possible to obtain the total horizontal displacement of the floor for each of the scale factors considered in the analysis. It is worth mentioning that the relative displacements of each story were calculated with respect to a base node of the mathematical model, which has restricted all types of displacement and rotation.

Finally, using Equation (1), inter-story drifts $\delta_{i}$ are calculated for each scale factor considered in the dynamic incremental analysis. The maximum value of the story drift is selected for each of them.

(1) $$\delta_{i}=\frac{\left|\Delta_{i}-\Delta_{i-1}\right|}{h_{i}}$$

where $\left|\Delta_{i}-\Delta_{i-1}\right|$ is the absolute value of the relative displacements of story i and i−1, and is the story height between levels i and i−1

The main criteria considered for the analysis are detailed below:

- To ensure better continuity in the IDA curve generated by the discrete points obtained for different intensity levels, scale increments between approximately 10% and 30% of the actual intensity value of the seismic event were used, depending on the maximum level of demand required to bring the structure to a limit state of collapse.
- Orthogonal components of the seismic records were assigned as input demand in each main direction of the structure (i.e., 14 components of each seismic event were used as demand in both the X and Y directions) to obtain the structure’s response with a higher degree of accuracy. This implies that a collapse brittleness curve will be obtained for each principal direction of the structure. Thus, the analysis was carried out considering the effect of each component on the numerical model independently.
- As previously stated, global dynamic instability occurs when a slight increase in the intensity of the seismic event results in a considerable increase in the structural response, i.e., when the IDA curve tends to flatten. The maximum value of IM that signals the beginning of structural collapse will be the one where the local tangent reaches 20% of the elastic slope, which is obtained when the building is subjected to a reduced scale accelerogram (for example, using a scale factor λ = 0.3).

The IDA curves were obtained using the standard procedure described previously and considering 5% damped spectral acceleration in the IM intensity measurement as a parameter, and evaluated for the first mode vibration period, i.e., IM= $S_{a}\left(T_{1},\;5\%\right)$ where $T_{1}$ is considered as the elastic fundamental period. It is widely recognized that referring to the elastic period usually leads to very dispersed IDA curves, so assuming that $S_{a}\left(T_{1},\;5\%\right)$ represents the IM can imply certain disadvantages due to the following: (i) the rejection of the higher modes of vibration, (ii) the elongation of the fundamental period associated with the non-linear hysteretic behavior of the structure, and (iii) the bias of the spectral shape that occurs when very high λ scale factors are used.

**Figure 6:**
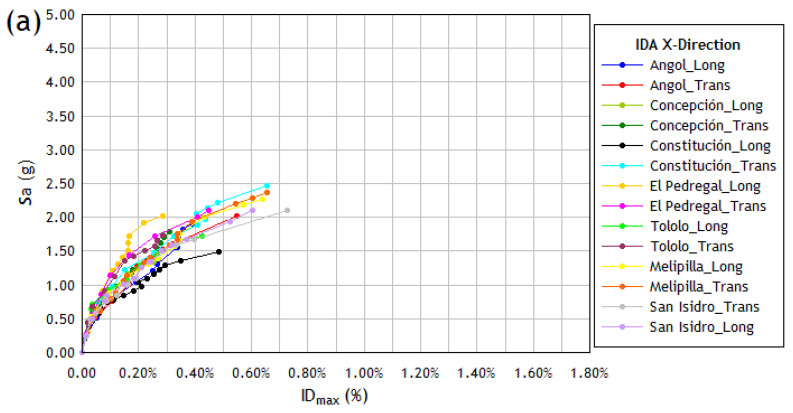

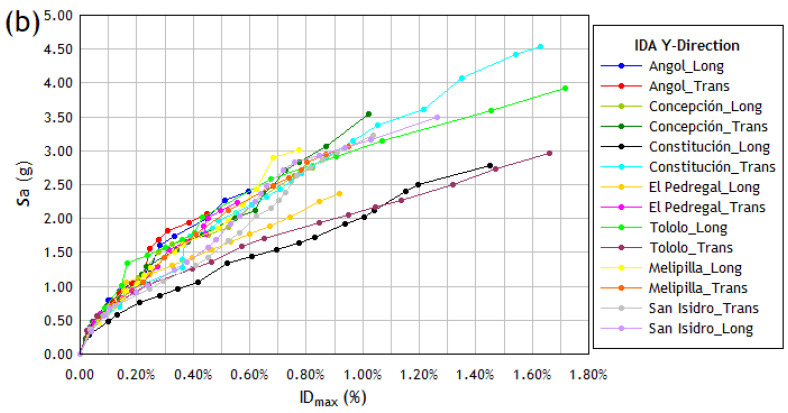
IDA curves with the maximum interstory drift (%) vs. spectral acceleration (g) obtained for the structural model (**a**) in the X direction and (**b**) in the Y direction, under the action of the 14 seismic records considered in the study.

Figure 6 depicts all the IDA curves of the building for each of the 14 seismic records considered as seismic demand in both X and Y directions. The discrete points of each graph are joined by a trend line to better visualize the behavior of the structure for each of the different factors used to scale the original intensity of each recording of accelerations, taking each analysis to levels of intensity in which displacements will be reached that indicate a collapse in the structure (values in which the analytical model is no longer considered reliable) or in which computational instability occurred. The general shape of the IDA curves is defined in [19] as a curve with strong softening. This form is one of the simplest and efficiently captures the point at which the collapse threshold defined in [19] is reached.

When subjected to strong ground motions, RC buildings reduce their resistance due to local cracking of the concrete and yielding of the reinforcing bars during reversals of the seismic load. This reduction leads to not only the dynamic modal characteristics of the system changing, but also their natural period when they are widely deformed in the plastic range, where the (apparent) inelastic period is usually longer than the elastic period (relevant for the undamaged structure) [63].

For a rigorous structural performance assessment, the inelastic period is a crucial parameter that can more effectively represent the IM intensity measure for IDA analysis than the fundamental elastic period. A recent study [64] proposed an alternative IDA procedure to reduce the high record-to-record variability of the curves representing the maximum demand in each building at different seismic intensity scales. In this case, the IM parameter is calculated employing the spectral acceleration (for a damping factor ξ=5%) considering the elongated inelastic period $T_{in}$ of the structure instead of the fundamental elastic period $T_{1}$, i.e., $IM=\left(T_{in},\;5\%\right)$, and where $T_{in}$ is determined as follows:

- Obtain the acceleration at the center of gravity of the building roof for each case from time–history analysis.
- Obtain the Fourier amplitude spectrum of the acceleration at the center of gravity of the roof and take the frequency $\omega_{in}$ of maximum spectral amplitude.
- Plot the intensity measure $IM=S_{a}\left(T_{in},\;5\%\right)$ against the damage indicator parameter DM to obtain the IDA curve, where the parameter chosen to quantify the damage to the structure (DM) is the maximum drift of the story $\delta_{max}$.

**Figure 7:**
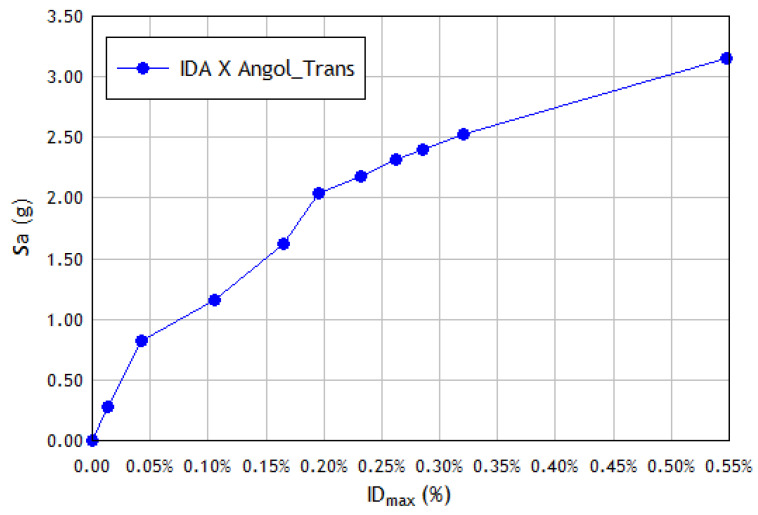
Alternative IDA curve in the X direction of the structural model, subjected to the transversal component of the seismic event recorded at Angol station.

**Figure 8:**
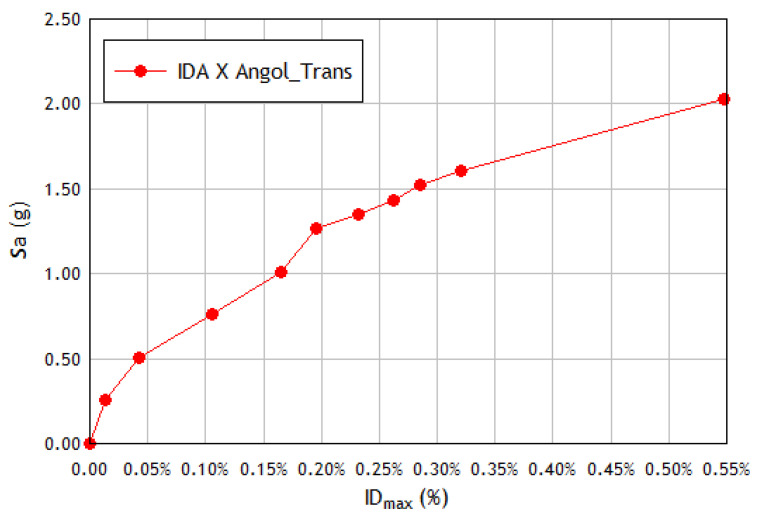
Standard IDA curve in the X direction of the structural model, subjected to the transversal component of the seismic event recorded at Angol station.

The IDA curve obtained using the alternative and the standard procedures are depicted in Figure 7 and Figure 8, respectively. The results obtained when applying the alternative procedure do not show improvement in reducing the dispersion of the IDA curves, nor do they show a clear tendency to reduce the ordinates of these curves. The ordinates of the IDA curves corresponding to stiff structures such as the one studied are not significantly reduced, as occurs with more flexible structures (frame structures) when the inelastic period is used in the calculation [64]. On the contrary, higher ordinate values are reached because the spectral ordinates are produced for these inelastic periods, so the application of this procedure is not considered advisable for very stiff structures.

Once the IDA curves have been obtained, a statistical analysis of the results is performed to define vulnerability functions that will serve to generate the fragility curves of the building.

For a state of damage other than null, the fragility curves follow a lognormal probability distribution, which allows defining each curve using two statistical parameters: a mean value and a dispersion value, with which a function can be established. Fragility is expressed as:

(2) $$P\left[d\geq d_{i}\right]=\phi \left[\frac{1}{\beta_{d_{i}}}\ln\left(\frac{IM}{IM_{d_{i}}}\right)\right]$$

where ϕ[•] is the cumulative standard normal distribution function, d is the expected damage level, IM is the variable that defines the measure of intensity. The values $IM_{d_{i}}$, $\beta_{d_{i}}$ and $P\left[d\geq d_{i}\right]$ represent the mean, dispersion measure and the probability that damage state d exceeds a discrete damage $d_{i}$ [65], respectively. The damage considered to obtain the fragility curves in this investigation is that which causes the imminent collapse of the structure and, as previously stated, is defined as the last point of the IDA curve with a tangent slope equal to or less than 20% of the elastic slope. There are several ways to obtain the fragility function’s statistical parameters, but for this work, the mean and dispersion values are determined according to FEMA P-58-1 [20] methodology.

Each point that defines collapse in an IDA curve is associated with a maximum inter-story drift $\delta_{max}$ and a collapse spectral acceleration $S_{a}\left(T_{1}\right)$, whose values differ for the 28 IDA curves obtained in the X and Y directions. The mean collapse capacity ${\hat{S}}_{a}\left(\bar{T}\right)$ in each direction corresponds to spectral acceleration $S_{a}\left(T_{1}\right)$ that is greater than 50% of the remaining values of intensity levels that cause collapse, i.e., if we order the spectral acceleration values $S_{a}\left(T_{1}\right)$ from low to high (14 in total for each direction of the structure) the average intensity of collapse ${\hat{S}}_{a}\left(\bar{T}\right)$ is located in the eighth position.

Table 3 and Table 4 show the values of maximum inter-story drift $\delta_{max}$, collapse spectral acceleration $S_{a}\left(T_{1}\right)$ and the scale factor λ for each accelerogram necessary to reach collapse in the direction X and Y, respectively.

According to the FEMA P-58-1 [20], the dispersions of demand parameters (story drift, maximum ground acceleration and velocity, axial forces, or other local actions in the building components, among others) are estimated based on the uncertainty inherent in the response calculation. Due to the potential inaccuracies in dispersion and correlation observed in small sets of analyses, the use of assumed uncertainty values is considered the best available information.

Two sources of uncertainty were considered in the calculation of the total dispersion β to define the collapse fragility curve. First, there is the uncertainty in the modeling $\beta_{m}$, as a result of inaccuracies in modeling of the elements, damping, and mass estimates. For the purposes of determining $\beta_{m}$, these inaccuracies have been associated with the level of building definition and construction quality assurance, $\beta_{c}$, and the quality and integrity of the non-linear analysis model, $\beta_{q}$. The total dispersion of the modeling $\beta_{m}$ can be estimated as follows:

(3) $$\beta_{m}=\sqrt{\beta_{c}^{2}+\beta_{q}^{2}}$$

Another source of uncertainty is the one associated with the variability of seismic records $\beta_{a\Delta }$. In structures that experience non-linear response, each record will produce somewhat different predictions of the maximum response quantities, resulting in record-to-record variability. Since the fundamental period of vibration of the structure in both directions is less than 0.2 s, a value of $\beta_{a\Delta }=0.45$ will be adopted conservatively. Then, the total dispersion β can be obtained using the following expression:

(4) $$\beta =\sqrt{{\beta_{a\Delta }}^{2}+({\beta_{c}}^{2}+{\beta_{q}}^{2})}=\sqrt{{\beta_{a\Delta }}^{2}+{\beta_{m}}^{2}}$$

Using the above information and substituting the values in Equation (4), a total dispersion value β=0.524 is obtained. Table 5 below summarizes the statistical parameters previously obtained for each orthogonal direction of the building under study.

From Equation (4), the cumulative standard normal distribution function ϕ[•] can be rewritten as:

(5) $$\phi \left[\frac{1}{\beta_{d_{i}}}\ln\left(\frac{IM}{IM_{d_{i}}}\right)\right]=\frac{1}{\beta \sqrt{2\pi }}\int_{-\infty }^{x}e^{\frac{{\left(u-{\hat{S}}_{a}\left(\bar{T}\right)\right)}^{2}}{2\beta^{2}}}du,x\;\epsilon \mathbb{R}$$

**Figure 9:**
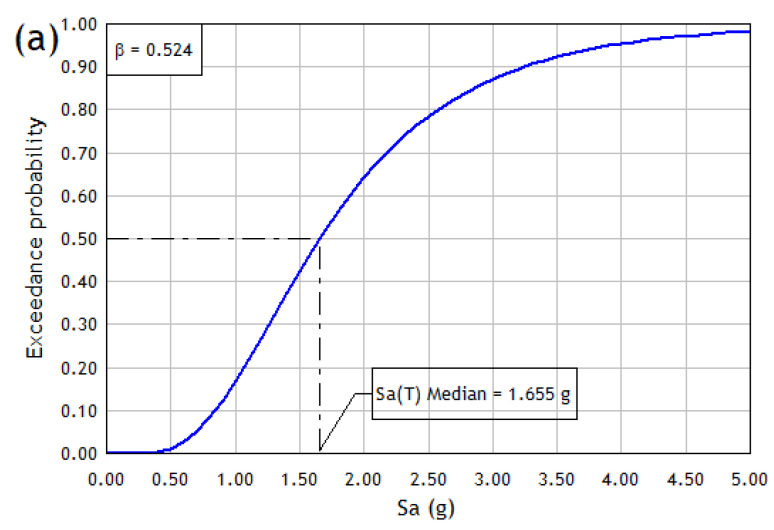

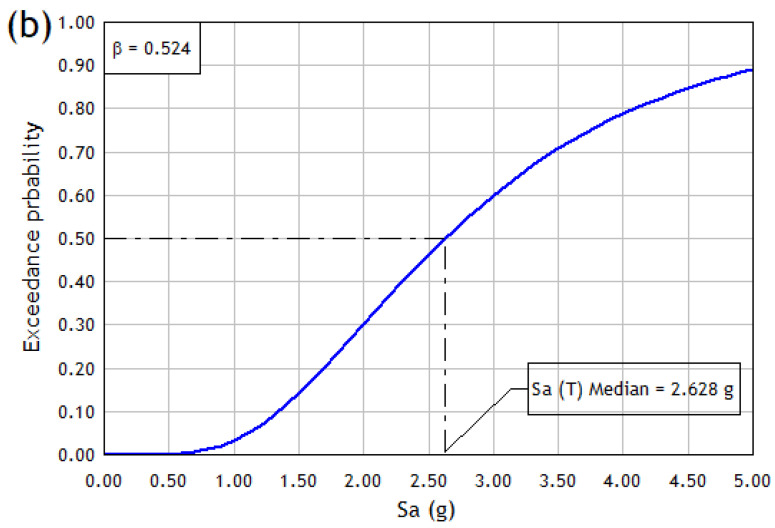
Collapse fragility curves in (**a**) X direction and (**b**) Y direction.

It can be seen that ϕ[•] can be defined as a function of the statistical parameters ${\hat{S}}_{a}\left(\bar{T}\right)$, β and values of spectral acceleration ranging from 0.1 g to a probability distribution of 100%. Therefore, using the results of the statistical parameters detailed in Table 5 and Equation (4) it is possible to obtain the collapse fragility curves of the structure in the X and Y directions, depicted in Figure 9. From this figure, it is possible to observe that the collapse fragility curve in the Y direction is more shifted to the right than the one obtained in the X direction since the average spectral acceleration of collapse ${\hat{S}}_{a}\left(\bar{T}\right)$, associated with a probability collapse of 50%, has a value of 1.655 g in the X direction and 2.628 g in the Y direction. This implies that, in a hypothetical case, if the structural model is subjected to a seismic event of similar intensity in both orthogonal directions and considering that the Y direction presents a ductile behavior, it will be less likely to reach a damage state of collapse. For example, if the model is subjected to a spectral acceleration of 1.5 g, the probability of reaching the collapse damage level would be 15% in the Y direction. In contrast, the same intensity in the X direction would have a probability of collapse of approximately 42%.

For buildings similar to the one under study, an adequate seismic response is not only associated with the achieved displacement control through stiffness, managed in the design process via the shear wall density but also requires an adequate ductility capacity so that the structure may be able to dissipate the energy entered by the earthquake, which is achieved by applying careful detailing of the structural members in the design stage [39,56].

## 5. Conclusions

The dynamic incremental analysis conducted to obtain the collapse fragility curves of the building, compared to a linear static or non-linear static analysis, provides more accurate or precise information to predict the structural behavior in the face of a given seismic demand. A set of records of earthquakes that occurred in the subduction zone of Chile have been used, which have introduced to the case study average acceleration values of 0.84 g and 1.18 g in the X and Y directions of the structure, respectively, which has allowed the proper application of dynamic incremental analysis.

The results show that the dynamic incremental analysis process is adequate to find the collapse threshold of very stiff structures like the one studied. The collapse thresholds of the IDA curves are high (1.655 g and 2.268 g in the X and Y directions, respectively). The difference in these values is due to the higher stiffness of the structure in the Y direction, compared to the X direction. If, in addition, the standard deviation is calculated using a procedure such as the FEMA P-58, collapse fragility curves can be generated for RC structures with shear walls, which have demonstrated a good behavior during recent earthquakes. These results allow us to compare the collapse fragility of this structural typology, which has shown adequate performance during the 2010 Maule earthquake, with other typologies. This allows for better understanding of structural behavior under destructive earthquakes and the functional relationship between a structure’s members and the possible sources of fragility that will allow decisions to be made in early stages of design or reinforcement in existing structures.

Based on the non-linear response history analysis results, it was possible to determine the maximum dynamic response of the structure as the intensity of the accelerograms used in this study increased. These obtained results, represented by IDA curves, are consistent and show that the structure reached acceptable drift values, thus demonstrating an adequate lateral resistance. Furthermore, it is concluded that the building responds with a higher level of stiffness and a lower deformation capacity prior to the collapse in the X direction and, therefore, shows a less ductile behavior. Conversely, the structure in the Y direction presents a higher level of ductility. Hence, it is possible to guarantee good seismic behavior of the structure.

Concerning the collapse fragility curves, it is determined that, for the same probability of reaching the whole damage level, a higher intensity measurement level is required in the Y direction than in the X direction. Consequently, this allows us to conclude that providing the system with a ductile failure mode generates a decrease in its seismic vulnerability to damage states associated with structural collapse. Regarding the shape of the curves, it is determined that they should have a smaller slope since, in this way, the system can resist a more significant variation of accelerations, thus being safer. The difference in stiffness between the two main directions of the structure means that the probability of collapse reached for a high level of acceleration (1.5 g) is 15% and 42% in the X and Y directions, respectively, evidencing the need for ensuring that the resulting design has a more balanced stiffness.

The alternative IDA procedure based on the inelastic period more effectively exposes the IM intensity measurement for the dynamic incremental analysis than the fundamental elastic period since the latter is only relevant for the undamaged structure and, therefore, does not reflect the progressive damage experienced by the structural system. On the other hand, according to the analyses performed, it is possible to infer that the alternative IDA procedure for very stiff buildings, as the structure under study, will not be as suitable as it is for frame buildings, where the IDA curve tends to be more flattened since when the structure is severely damaged, the inelastic period is quite long and, therefore, spectral acceleration values are obtained within the descending branch of the elastic response spectrum.

The results obtained allow a comparison with those of other structural typologies, designed with international codes. It is suggested that in future work the typology studied can be compared with buildings designed with said codes to check the collapse thresholds reached.

## Figures and Tables

**Table 1:** Characteristics of the acceleration records chosen for the analyses.

Earthquake	Date	Magnitude (Mw)	Coord. Epicenter	Station	Component	PGA (g)	Significative Duration (s)
Valparaiso	03-03-85	8.0 (CSN)	33.240° S	Melipilla	Transv.	0.496	19.82
					Long.	0.499	18.80
		7.4 (USGS)	71.850° W	San Isidro	Transv.	0.532	36.20
					Long.	0.503	39.80
Bio-Bio	27-02-2010	8.8 (CSN)	36.290° S	Angol	Transv.	0.446	45.89
					Long.	0.523	44.84
				Concepcion	Transv.	0.617	56.49
		8.8 (USGS)	73.239° W		Long.	0.466	59.25
				Constitucion	Transv.	0.415	38.43
					Long.	0.473	51.42
Coquimbo	16-09-2015	8.4 (CSN)	31.553° S	El Pedregal	Transv.	0.557	36.43
					Long.	0.504	33.02
		8.3 (USGS)	71.864° W	Tololo	Transv.	0.470	42.02
					Long.	0.412	43.30

**Table 2:** First ten modes characteristic of the building.

Mode	Period [s]	Frequency [Hz]	Participative Mass [%]					
			Ux	Uy	Uz	Rx	Ry	Rz
1	0.169	5.904	1.87	43.23	0.00	25.59	0.62	15.96
2	0.153	6.552	4.06	20.07	0.10	12.64	1.01	41.82
3	0.090	11.058	56.46	0.00	0.06	0.00	17.24	5.14
4	0.087	11.536	0.06	0.04	0.00	0.04	0.02	0.01
5	0.051	19.655	0.01	0.33	14.26	4.03	6.62	0.09
6	0.050	19.965	0.09	0.03	2.03	0.15	0.19	0.02
7	0.050	19.989	0.08	0.23	5.28	1.59	0.54	0.02
8	0.049	20.553	0.21	0.00	7.88	1.76	8.47	0.01
9	0.047	21.393	0.61	0.10	0.06	0.48	0.62	0.34
10	0.046	21.937	0.00	0.00	0.54	0.00	0.31	0.11

**Table 3:** Values of maximum drift of story $\delta_{max}$, spectral acceleration $S_{a}\left(T_{1}\right)$ and collapse scale factor λ for the IDA curves obtained in the Y direction of the structure.

IDA X	Scale Factor λ	$\delta_{máx\;\left(\%\right)}$	$S_{a}{\left(T_{1}\right)}_{\left(g\right)}$
Constitución_Long	1.6	0.199	1.036
Tololo_Long	1.6	0.164	1.151
Concepción_Long	1.3	0.171	1.159
San_Isidro_Trans	1.5	0.210	1.257
ElPedregal_Transv	1.5	0.168	1.433
San_Isidro_Long	1.8	0.281	1.511
Angol_Transv	1.8	0.285	1.521
Tololo_Transv	2.2	0.265	1.655
Concepción_Transv	2.1	0.290	1.705
Melipilla_Long	2.0	0.344	1.748
Angol_Long	2.1	0.358	1.824
ElPedregal_Long	1.9	0.218	1.918
Melipilla_Transv	2.2	0.387	1.933
Constitución_Transv	2.5	0.403	2.049

**Table 4:** Values of maximum drift of story $\delta_{max}$, spectral acceleration $S_{a}\left(T_{1}\right)$ and collapse scale factor λ for the IDA curves obtained in the Y direction of the structure.

IDA Y	Scale Factor λ	$\delta_{máx\;\left(\%\right)}$	$S_{a}{\left(T_{1}\right)}_{\left(g\right)}$
Tololo_Transv	0.5	0.060	0.569
ElPedregal_Long	0.9	0.161	1.065
Angol_Transv	1.4	0.308	1.813
ElPedregal_Transv	1.8	0.498	2.122
Angol_Long	1.7	0.514	2.270
Constitución_Long	2.6	1.198	2.498
Tololo_Long	2.3	0.675	2.578
Concepción_Long	2.1	0.761	2.628
Concepción_Transv	2.3	0.727	2.716
San_Isidro_Long	2.5	0.759	2.823
Melipilla_Transv	2.4	0.804	2.835
Melipilla_Long	2.5	0.684	2.895
San_Isidro_Transv	2.5	0.910	2.983
Constitución_Transv	3.5	1.349	4.067

**Table 5:** Parameters obtained for the threshold state of collapse of the structure in the X and Y directions.

Direction X		Direction Y	
${\hat{S}}_{a}\left(\bar{T}\right)(g)$	β	${\hat{S}}_{a}\left(\bar{T}\right)(g)$	β
1.655	0.524	2.628	0.524

## Data Availability

The relevant data from this research are available in the authors’ repositories.
